# Association of *FCGR2A* rs1801274 polymorphism with susceptibility to autoimmune diseases: A meta-analysis

**DOI:** 10.18632/oncotarget.9831

**Published:** 2016-06-05

**Authors:** Chang'e Zhang, Wenju Wang, Hong'e Zhang, Lulu Wei, Shuping Guo

**Affiliations:** ^1^ Department of Dermatology, Zhengzhou Children's Hospital, Henan, China; ^2^ Department of Dermatology, The Second People's Hospital in Chengdu, Sichuan, China; ^3^ Department of Medicine, Xiangfu District Hospital of Traditional Chinese Medicine, Kaifeng, Henan, China; ^4^ Department of Dermatology, The First Affiliated Hospital, Shanxi Medical University, Taiyuan, Shanxi, China

**Keywords:** autoimmune diseases, FCGR2A, polymorphism, susceptibility, meta-analysis, Immunology and Microbiology Section, Immune response, Immunity

## Abstract

**Objectives:**

The aim of this meta-analysis was to estimate the association between the *FCGR2A* rs1801274 polymorphism and the susceptibility to autoimmune diseases more precisely.

**Methods:**

A meta-analysis was conducted on the association between the *FCGR2A* gene variants and ADs by allelic contrast, homozygote contrast, the recessive model, and the dominant model.

**Results:**

A total of 17 studies with 30 comparisons in different populations and genotype-methods were available for this meta-analysis, including 10 Kawasaki disease (KD), 7 Ulcerative colitis (UC), 6 Crohn's disease (CD), 3 Rheumatoid arthritis (RA), 2 Systemic lupus erythematosus (SLE), 1 Autoimmune thyroid disease (ATD) and 1 diabetes mellitus type 1 (T1D). A significant association between *FCGR2A* rs1801274 polymorphism were found in KD (OR = 1.409, *P* < 0.001) and UC (OR = 1.237, *P* < 0.001). A overall meta-analysis increased risk of AD significant association between *FCGR2A* rs1801274 gene polymorphism and ADs under allelic (OR = 1.378, *P*=0.000), homozygous (OR: 1.866, *P*=0.001), dominant (OR = 1.667, *P* = 0.000) and recessive (OR = 1.434, *P*=0.000) in Asian population. Meanwhile, a decreased risk of AD was detected in the allelic (OR= 0.882, *P* = 0.011), homozygous (OR = 0.777, *P* = 0.013), dominant (OR = 0.850, *P* = 0.032) and recessive (OR = 0.840, *P* = 0.048) in African-American population.

**Conclusions:**

This meta-analysis demonstrates that the *FCGR2A* rs1801274 G-allele confers susceptibility to KD and UC. Data also suggests that the *FCGR2A* rs1801274 polymorphism may be associated with the susceptibility of multiple ADs in Asian and African-American populations.

## INTRODUCTION

Autoimmune diseases (ADs) are some complex disorders includes a substantial portion of pathobiology, the potential inheritance and induced environment, efficacy of the common therapies and co-occurrence among diseases. ADs accounted for 4-5% of the population and had always become difficult issues [1]. More recently, genome-wide association study (GWAS) and the sequencing approaches had been widely used to discover low-frequency disease risk alleles, and thousands susceptibility locis might predispose to multiple ADs. An expected observation for risk variants are shared across diseases. Colocalized genetic effects had enlighted a common pathway to interfere with pathobiology underlying defect immune functions [2, 3]. Several genes are associated in multiple ADs and are included in the shared network [3, 4].

The studied variant rs1801274 in *FCGR2A* had been reported and it was associated with the susceptibility to multiple autoimmune diseases, including systemic lupus erythematosus (SLE), Kawasaki disease (KD), diabetes mellitus type 1 (T1D), autoimmune thyroid disease (ATD), ulcerative colitis (UC), Crohn's disease (CD), rheumatoid arthritis (RA) [5–11], etc. However, the inadequate statistical data, ethnic differences or publication bias resulting in the controversial and inconclusive in different case-control studies. Here, we performed a meta-analysis to evaluate between *FCGR2A* rs1801274 polymorphisms and the susceptibility to multiple ADs.

## RESULTS

### Studies included in the meta-analysis

A total of 43 relevant studies with the *FCGR2A* rs1801274 polymorphism and autoimmune diseases were identified through PubMed and *Web of Science* search, and 17 articles included KD [6, 16–21], UC [9, 10, 22, 23], CD [7, 10, 22, 24], ATD [8], RA [7, 25], T1D [7] and SLE [26, 27] met the inclusion criteria for analysis. And 8 articles included multiple case-control studies in different autoimmune diseases or different populations. Overall, 30 eligible case-control comparisons including 16760 ADs and 30585 controls were enrolled in the meta-analysis. The ethnicities encompassed in qualified studies were stratified into Caucasian, non-Caucasian European, African-American, and Asian populations. The characteristics of the selected studies were summarized in (Supplementary Table S1)

The results revealed significant association between *FCGR2A* rs1801274 A-allele and KD (OR = 1.409, 95% CI: 1.320-1.505, *P* < 0.001) and UC (OR = 1.237, 95% CI: 0.968-1.581, *P* < 0.001). In KD, significant association were observed in the population of Caucasian (OR = 1.466, 95% CI: 1.276-1.685, *P* < 0.001) and Asia population(OR = 1.395, 95% CI: 1.285-1.515, *P* < 0.001). For UC, significant association was found in Asia population (OR = 1.480, 95% CI: 1.356-1.615, *P* < 0.001) (Table 1).

**Table 1:** Meta-analysis of the FCGR2A rs1801274 polymorphism in autoimmune diseases

Diseases	Comparison	Population	Sample size	Study number	Test of association (A *vs* G)			Model	Test of heterogeneity		
					OR (95% CI)	Z	*P* Value		Q	*P* Value	I2 (%)
KD	A *vs* G	Overall	17865	10	1.409(1.320-1.505)	10.23	<0.001	R	9.53	0.39	5.6
		Caucasian	6754	2	1.466(1.276-1.685)	5.39	<0.001	R	0.10	0.751	0
		Asian	11111	8	1.395(1.285-1.515)	7.93	<0.001	R	9.05	0.249	22.6
UC	A *vs* G	Overall	9752	7	1.237(0.968-1.581)	3.50	<0.001	R	34.91	0	82.8
		Caucasian	3238	5	1.059(0.959-1.169)	1.13	0.257	NA	0	NA	NA
		Asian	5655	1	1.480(1.356-1.615)	8.80	<0.001	F	2.80	0.592	0
		Non-Caucasian Europe	859	1	0.969(0.801-1.171)	0.33	0.742	NA	0	NA	NA
CD	A *vs* G	Overall	8136	6	0.832(0.592-1.168)	1.06	0.288	R	119.99	0	95.8
		Caucasian	5578	3	0.709(0.363-1.382)	1.01	0.312	R	115.24	0	98.3
		Non-Caucasian Europe	1324	2	0.937(0.804-1.093)	0.83	0.407	F	0.10	0.747	0
		Asian	1234	1	1.043(0.867-1.253)	0.44	0.657	NA	0.00	NA	NA
RA	A *vs* G	Overall	2278	3	1.008(0.493-2.061)	0.02	0.983	R	50.43	0	96.0
		Caucasian	1856	2	0.875(0.340-2.252)	0.28	0.782	R	37.42	0	97.3
		Asian	422	1	1.358(0.965-1.909)	1.76	0.079	NA	0	NA	NA
SLE	A *vs* G	Overall	4366	2	0.941(0.864-1.025)	1.39	0.165	R	7.53	0.06	86.7
	AA *vs* GG	Overall	4366	2	0.886(0.743-1.057)	1.34	0.179	R	8.28	0.004	87.9
	AA + AG *vs* GG	Overall	4366	2	0.914(0.797-1.049)	1.28	0.200	R	6.55	0.01	84.7
	AG + GG *vs* AA	Overall	4366	2	1.072(0.932-1.234)	0.97	0.330	R	4.22	0.04	76.3

KD, Kawasaki disease; UC, Ulcerative colitis; CD, Crohn's disease; RA, rheumatoid arthritis; SLE, Systemic lupus erythematosus; HWE, Hardy–Weinberg equilibrium; OR, odds ratio; CI, confidence interval; F, fixed-effects model; R, random-effects model; NA, not applicable.

The summary of meta-analysis for the *FCGR2A* rs1801274 polymorphism with autoimmune diseases was shown in Table 2. The results were showed no significant association between *FCGR2A* rs1801274 with susceptibility to these all phenotypes in overall populations. Additionally, sub-group analysis were also performed in the study. In Asian population, increased risk of ADs in the allelic (OR = 1.378, 95% CI: 1.287-1.475, *P* = 0.000), homozygous (OR = 1.866, 95% CI: 1.631-2.135, *P* = 0.001), dominant (OR = 1.667, 95% CI: 1.462-1.901, *P* = 0.000) and recessive (OR = 1.434, 95% CI: 1.319-1.559, *P* = 0.000) were found. In African-American population, decreased risk of AD was detected in the allelic (OR = 0.882, 95% CI: 0.800-0.972, *P* = 0.011), homozygous (OR = 0.777, 95% CI: 0.638-0.947, *P* = 0.013), dominant (OR = 0.850, 95% CI: 0.733-0.986, *P* = 0.032) and recessive (OR = 0.840, 95% CI: 0.707-0.999, *P* = 0.048). However, no significant association was found when all the contrasts were performed in Caucasian and non-Caucasian Europe populations.

**Table 2:** Meta-analysis of the association between the FCGR2A rs1801274 polymorphism and multiple autoimmune diseases

Comparison	Population	No. of studies	Test of association (A *vs* G)			Model	Test of heterogeneity		Publication bias	
			OR (95% CI)	Z	*P* Value		*P* Value	I2 (%)	Begg	Egger
A *vs* G	Overall	30	1.119(0.984-1.272)	1.71	0.086	R	0	94.2	0.363	0.148
	Caucasian	10	0.843(0.653-1.088)	1.31	0.189	R	0	96.9	0.592	0.542
	Asian	16	1.378(1.287-1.475)	9.23	0	R	0.034	43.3	0.471	0.132
	Non-Caucasian Europe	3	0.949(0.843-1.070)	0.85	0.395	F	0.916	0	1	0.581
	African-American	1	0.882(0.800-0.972)	2.53	0.011	NA	NA	NA	NA	NA
AA *vs* GG	Overall	30	1.237(0.968-1.581)	1.70	0.090	R	0	92.0	0.232	0.068
	Caucasian	10	0.745(0.466-1.192)	1.23	0.220	R	0	96.2	0.592	0.579
	Asian	16	1.866(1.631-2.135)	9.08	0	F	0.751	0	0.322	0.071
	Non-Caucasian Europe	3	0.901(0.71-1.145)	0.85	0.396	F	0.917	0	1	0.585
	African-American	1	0.777(0.638-0.947)	2.50	0.013	NA	NA	NA	NA	NA
AA + AG *vs* GG	Overall	30	1.186(0.998-1.409)	1.94	0.052	R	0	87.1	0.148	0.036
	Caucasian	10	0.839(0.613-1.148)	1.10	0.272	R	0	93.8	0.858	0.640
	Asian	16	1.667(1.462-1.901)	7.64	0.000	F	0.97	0	0.126	0.118
	Non-Caucasian Europe	3	0.933(0.764-1.139)	0.68	0.497	F	0.946	0	1	0.602
	African-American	1	0.850(0.733-0.986)	2.15	0.032	NA	NA	NA	NA	NA
AG + GG *vs* AA	Overall	30	1.115(0.949-1.311)	1.32	0.186	R	0.000	92.6	0.532	0.649
	Caucasian	10	0.780(0.552-1.102)	1.41	0.159	R	0.000	95.9	0.858	0.535
	Asian	16	1.434(1.319-1.559)	8.45	0.000	R	0.032	43.7	0.589	0.257
	Non-Caucasian Europe	3	0.933(0.7721.129)	0.71	0.478	F	0.941	0	1	0.563
	African-American	1	0.840(0.707-0.999)	1.97	0.048	NA	NA	NA	NA	NA

HWE, Hardy–Weinberg equilibrium; OR, odds ratio; CI, confidence interval; F, fixed-effects model; R, random-effects model; NA, not applicable.

### Test of heterogeneity

As shown in Table 1, the *P* value for the distribution of genotype for HWE in the control groups was calculated. Only one study in control population was not consistent with HWE. However, when studies were omitted individually from the meta-analysis to evaluate possible individual influences, heterogeneity disappear in CD and RA.

Then we pooled results according to the ethnicity of study populations. Heterogeneity was found for the *FCGR2A* rs1801274 A-allele and CD in Caucasian population. The study with the highest OR were then excluded, and the heterogeneity disappeared but the result remained none significant (OR = 1.067, 95% CI: 0.995-1.143, *P* = 0.258).

### Publication bias

Publication bias analysis on KD, UC, CD and overall phenotypes were performed. but not on RA, SLE, ATD and T1D due to small numbers of these studies. No obvious asymmetry evidence was detected according to the shapes of the funnel plots (data not shown).

No evidence of publication bias by the method of Egger's test and Begg's tests (Table 2) were found. However, there was evidence of heterogeneity in this study. Assessment of potential sources of heterogeneity, by both stratified and meta-regression analyses, found that sample size, ethnicity, publication year or disease phenotype was unable to explain the variance. The results suggested that these potential factors were probably not the major sources of heterogeneity (data not shown).

## DISCUSSION

ADs invokeed a wide spectrum of signs and symptoms, genetic analysis revealed multiple genes underlying distinct autoimmune conditions, which provided direct evidence that might have common immunopathologic mechanism.

*FCGR2A* gene was located on chromosome 1q23, which consists of 7 exons spanning approximately 18.58 kb of genomic DNA. It encodes a member of a family of Fc γ receptors for immunoglobulin G. *FCGR2A* protein played an essential role in the protection against foreign antigens by removing antigen-antibody complexes in the circulation, and transduces activating signals into cells *via* immune receptor when ligated with immune complexes [28]. This stimulatory receptor was expressed by most leukocytes, including monocytes, dendritic cells, macrophages, natural killer cells, platelets and endothelial cells, and a subpopulation of T-cells. Upon binding of antibodies or autoantibodies, *FCGR2A* and *FCGR3A* activate immune cell functions, including phagocytosis, and the release of inflammatory mediators, were therefore linked to the pathogenic consequences which triggered by autoantibodies or immune complexes in multiple immune-mediated diseases [29].

**Figure 1:**
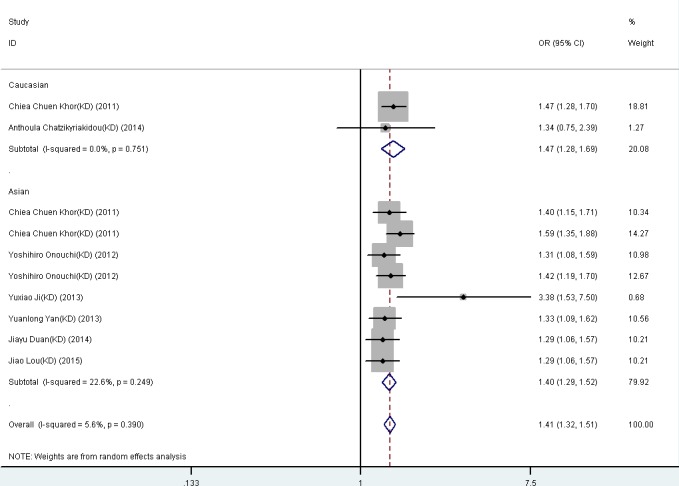
Forest plot for the meta-analysis of association between *FCGR2A* rs1801274 polymorphism and kawasaki disease (KD) stratified by the ethnicity of study population ORs and 95% CIs for the outcomes of the allelic comparison in the overall population (***P*** < 0.001, OR = 1.41, 95% CI = 1.32-1.51) and Caucasians (***P*** < 0.001, OR = 1.47, 95% CI = 1.28-1.69).

The associated polymorphism *FCGR2A* rs1801274 was a missense variant leading to an amino-acid substitution of histidine by arginine at position 131 (H131R). The variant H131R was reported to interact differently with immunoglobulin G subclasses, and associated with the susceptibility to multiple autoimmune diseases. Specifically, the substitution of *FCGR2A* rs1801274 affected the Fc region of IgG receptor and determined the affinity of *FCGR2A* for IgG subclasses. This variant was capable of binding to and mediating phagocytosis with IgG2, potentially leading to altered immune response to infectious agents and activation of B cells and overproduction of cytokines [30].

However, owing to low prevalence or the inadequate statistical power, the discrepancy of the results was always achieved among different studies. Meta-analysis was a method of increasing the effective sample size through the pooling of datas from individual studies, thus enhancing the statistical power of the analysis for the estimation of genetic effect [31].

The results of the meta-analysis revealed significant association between the *FCGR2A* rs1801274 polymorphism and autoimmune diseases including KD and UC, indicating a protecting effect to KD and predisposing to UC, *FCGR2A* rs1801274 polymorphism might have no effect on CD or RA. However, the exact biological mechanism that the *FCGR2A* gene polymorphisms influence susceptibility to ADs remains unclear.

**Figure 2:**
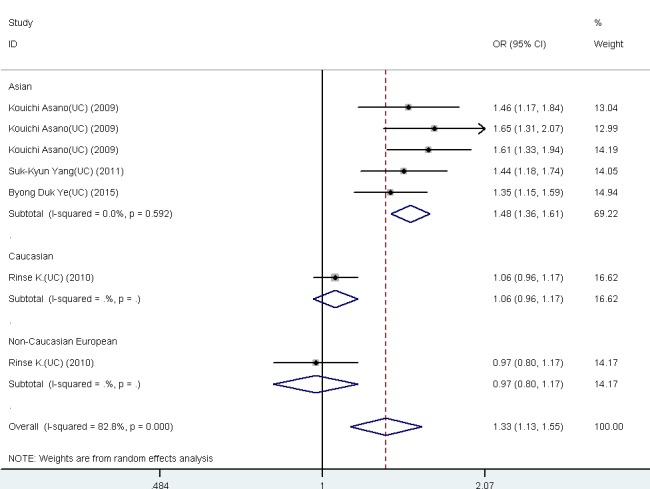
Forest plot for the meta-analysis of association between *FCGR2A* rs1801274 polymorphism and ulcerative colitis (UC) ORs and 95% CIs for the outcomes of the allelic comparison in the overall population (***P*** < 0.001, OR = 1.33, 95% CI = 1.13−1.55) and Asian (***P*** < 0.001, OR = 1.48, 95% CI = 1.36−1.61).

The significant association between *FCGR2A* rs1801274 polymorphism susceptibility to ADs in the overall population was failed to detect. Sub-population analysis revealed association between different genotypes and Asia population. The AD risk increased significantly in Asia populations using allelic, homozygous, recessive and dominant genetic models. Further studies including larger sample size, well-designed case-control studies in different ethnic populations will be needed to unravel their exact roles in the pathogenesis of multiple ADs.

Several limitations of the present meta-analysis should be showed. First, for several autoimmune diseases, the number of studies is small, and this might cause insufficient power to detect slight association. Second, significant heterogeneity between-study was found in some comparisons. Third, the majority of the included studies were conducted in population of Caucasian and Asia, thus further studies in other ethnic populations were required. Fourth, the present meta-analysis was based on uncorrected estimates. A more precise analysis could be performed if the potential confounding factors including sex, age, environmental factors and other lifestyle factors were available. Thus, the results of the meta-analysis should be interpreted with caution.

**Figure 3:**
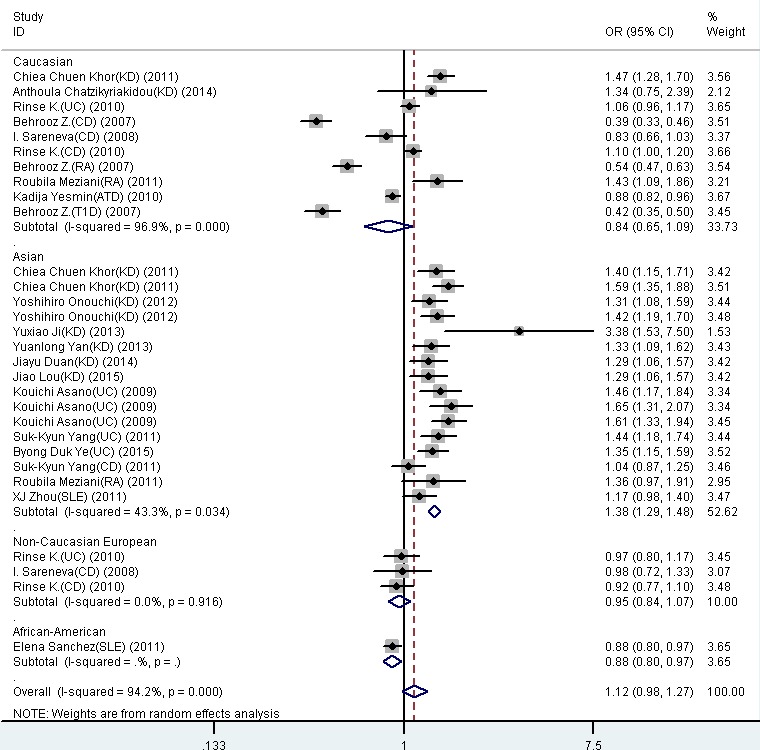
Forest plot for the meta-analysis of association between *FCGR2A* rs1801274 polymorphism and autoimmune diseases stratified by the ethnicity ORs and 95% CIs for the outcomes of the allelic comparison in the overall population (***P*** = 0.086, OR = 1.12, 95% CI = 0.98-1.27) and Asian (***P*** < 0.001, OR = 1.38, 95% CI = 1.29-1.48).

In summary, the meta-analysis demonstrated that the *FCGR2A* rs1801274 polymorphism was associated with the susceptibility to multiple autoimmune diseases including KD and UC. It also provided evidence that the *FCGR2A* rs1801274 polymorphism may be associated with susceptibility to multiple ADs in Asia population. The *FCGR2A* rs1801274 polymorphism in multiple autoimmune diseases provides further evidence supporting the concept of common gene underlying multiple autoimmune diseases. Further studies including larger sample size, well-designed case-control studies in different ethnic populations will be needed to unravel their exact roles in the pathogenesis of multiple ADs.

## MATERIALS AND METHODS

A comprehensive search examining the association between the *FCGR2A* rs1801274 polymorphism with autoimmune diseases were conducted. by searching the following Medical Subject Heading (MeSH) terms and/or text words: “Fc fragment of IgG, low-affinity IIa, receptor”, “*FCGR2A*”, “polymorphism”, “autoimmune diseases”, and “autoimmunity” in PubMed and *Web of Science* literature base for the period up to 2015-12-01, and relevant articles were identified for further literature filtering. A study was included if the following criteria were satisfied (1) case-control genetic association study published before June 2015; (2) genotype frequencies in the cases group and controls group were both available for estimating an odds ratios (OR) and their 95% confidence interval (CI). The exclusion criteria included: (1) studies that contained overlapping data with other literatures; (2) studies which the genotype or allele frequency could not be obtained; (3) data came from case-reports, reviews or abstracts; (4) control group did not confirm to Hardy-Weinberg equilibrium (HWE).

The following information from each study were extracted: first author's name, publication year, country, ethnicity, genotype-methods, the number of cases and controls and allele frequencies of the *FCGR2A* rs1801274 polymorphism. Data extraction was performed independently by two authors, and disagreements were solved by discussion.

Statistical analysis for the meta-analysis was conducted by Stata version 10.0 (Stata Corporation, College Station, TX). This meta-analysis examined the contrast of G *versus* A (allelic contrast), GG *versus* AA (homozygote contrast), G/G+G/A *versus* AA (dominant models), G/G *versus* A/A+G/A (recessive models). The heterogeneity between studies was evaluated by Cochrane's Q-statistic as well as I^2^-statistic which was used to quantify the effect of heterogeneity (I^2^ = 100% (Q-df)/Q) [12]. Subgroup analysis stratified by ethnicity were also performed in this meta-analysis. Meta-regression analysis was undertaken to explore potential sources of heterogeneity across studies when statistical heterogeneity was detected.

The funnel plot was used to assess potential publication bias [13]. Egger's test and Begg's tests were performed to evaluate potential publication bias of the literatures [14, 15]. The significance of the intercept was calculated by the *t*-test suggested by Egger, *P* value less than 0.05 was considered significant publication bias.
